# Health related quality of life six months following surgical treatment for secondary peritonitis – using the EQ-5D questionnaire

**DOI:** 10.1186/1477-7525-5-35

**Published:** 2007-07-02

**Authors:** Kimberly R Boer, Oddeke van Ruler, Johannes B Reitsma, Cecilia W Mahler, Brent C Opmeer, E Ascelijn Reuland, Hein G Gooszen, Peter W de Graaf, Eric J Hesselink, Michael F Gerhards, E Philip Steller, Mirjam A Sprangers, Marja A Boermeester, Corianne A De Borgie, The Dutch Peritonitis Study Group

**Affiliations:** 1 Department of Clinical Epidemiology, Biostatistics and Bioinformatics, Academic Medical Center, Amsterdam, The Netherlands; 2 Department of Surgery, Academic Medical Center Amsterdam, The Netherlands; 3 Department of Surgery, University Medical Center Utrecht, Utrecht, The Netherlands; 4 Department of Surgery, Reinier de Graaf Hospital, Delft, The Netherlands; 5 Department of Surgery, Gelre Hospital, Apeldoorn, The Netherlands; 6 Department of Surgery, Onze Lieve Vrouwe Gasthuis, Amsterdam, The Netherlands; 7 Department of Surgery, Sint Lucas Andreas Hospital, Amsterdam, The Netherlands; 8 Department of Medical Psychology, Academic Medical Center Amsterdam, The Netherlands; 9 Department of Surgery, Academic Medical Center Amsterdam, The Netherlands

## Abstract

**Background:**

To compare health related quality of life (HR-QoL) in patients surgically treated for secondary peritonitis to that of a healthy population. And to prospectively identify factors associated with poorer (lower) HR-QoL.

**Design:**

A prospective cohort of secondary peritonitis patients was mailed the EQ-5D and EQ-VAS 6-months following initial laparotomy.

**Setting:**

Multicenter study in two academic and seven regional teaching hospitals.

**Patients:**

130 of the 155 eligible patients (84%) responded to the HR-QoL questionnaires.

**Results:**

HR-QoL was significantly worse on all dimensions in peritonitis patients than in a healthy reference population. Peritonitis characteristics at initial presentation were not associated with HR-QoL at six months. A more complicated course of the disease leading to longer hospitalization times and patients with an enterostomy had a negative impact on the mobility (p = 0.02), self-care (p < 0.001) and daily activities: (p = 0.01). In a multivariate analysis for the EQ-VAS every doubling of hospital stay decreases the EQ-VAS by 3.8 points (p = 0.015). Morbidity during the six-month follow-up was not found to be predictive for the EQ-5D or EQ-VAS.

**Conclusion:**

Six months following initial surgery, patients with secondary peritonitis report more problems in HR-QoL than a healthy reference population. Unfavorable disease characteristics at initial presentation were not predictive for poorer HR-QoL, but a more complicated course of the disease was most predictive of HR-QoL at 6 months.

## Background

Secondary peritonitis has a high in-hospital mortality (24–35%), continued high post-hospital discharge mortality, as well as a considerable long-term morbidity [1-5]. Patients are hospitalized for extensive periods of time and often endure lengthy intensive care unit (ICU) stays [5-12].

Recently, improving Health Related Quality of Life (HR-QoL) in patients with sepsis [11,13,14] has become a complementary goal in patient care [15]. The importance of HR-QoL will continue to grow with improvement in peritonitis survival. Till now, most HR-QoL data in secondary peritonitis and abdominal sepsis have been collected retrospectively [4,13,14,16,17]. These studies have shown that peritonitis patients suffer from HR-QoL impairments both in the short-term as well as the long-term. Good quality data from prospective studies are necessary to identify factors related to lower HR-QoL. Insight into these factors is needed to inform patients, to develop preventive measures for high-risk patients, and to provide tailored support for individual patients.

The aims of this study were twofold. Firstly, to assess HR-QoL in patients with secondary peritonitis, and to compare this with HR-QoL reported for a general reference population [18]. And secondly, to determine which factors (patient, peritonitis and postoperative) are related to HR-QoL six months following patients with severe secondary peritonitis (APACHE II > 10)[19,20].

## Methods

### Study design

This study was embedded in an ongoing peritonitis trial evaluating two surgical strategies for patients with peritonitis, initiated by the Academic Medical Center (AMC), Amsterdam, The Netherlands. Patients were enrolled between December 2001 and August 2005 in 2 academic and 7 regional teaching hospitals in The Netherlands.

### Patients

Patients were eligible for the RELAP trial if they had a clinical diagnosis of secondary peritonitis requiring emergency laparotomy. Peritonitis had to be caused by perforation or infection of a visceral organ, or ischemia/necrosis of part of the gastrointestinal tract or postoperative peritoneal infection. An Acute Physiology And Chronic Health Evaluation (APACHE)-II score above 10 was required, as the preferred strategy for mild peritonitis (APACHE-II score ≤ 10) is on-demand. Exclusion criteria included: age below 18 or above 80; peritonitis due to bowel perforation after endoscopy operated within 24 hours; abdominal infection due to indwelling dialysis (CAPD) catheter; acute pancreatitis; expected survival of less than 6 months due to disseminated malignancy; severe brain damage due to trauma or anoxia; imperative relaparotomy (gauze packing).

**Figure 1:**
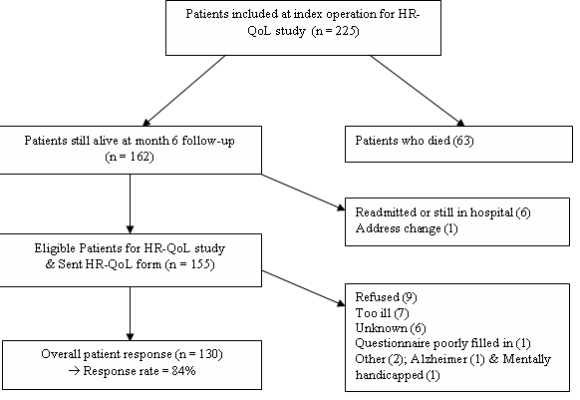
Flowchart summarizing inclusion and response.

To be eligible for participation in the present HR-QoL study, patients had to be alive and out of hospital at six months following index laparotomy (Figure 1).

### Instruments

HR-QoL was assessed approximately six months after the index laparotomy by administering the patient self-report Euroqol 5-Dimensions (EQ-5D) question which includes five dimensions and the Euroqol-Visual Analogue Scale (EQ-VAS) [21]. The Euroqol instruments have been extensively validated, including Dutch healthy individuals, and were recently recommended as the instrument of choice in critical care studies [22-25]). EQ-5D was originally designed to complement other instruments but is now increasingly used as a 'stand alone' measure.

The EQ-5D measures five health dimensions: mobility (MO), self-care (SC), daily activities (DA), pain/discomfort (PD), and mood (MD) consisting of both anxiety and depression. In the EQ-5D patients report: 0 (no problems), 1 (moderate problems), and 2 (extreme problems) [26]. Whilst the EQ-VAS is a thermometer-like scale, in which patients rate their overall well-being from 0 (worst imaginable overall health) to 100 (best imaginable overall health) [26,27].

#### Data collection

Preoperative risk factors and postoperative morbidity data were prospectively collected for all eligible patients. HR-QoL data were collected six months after index operation. EQ-5D and EQ-VAS questionnaires were sent by mail to patients who survived at least six months, with a reminder by phone within two weeks if there was no response. After one month without response patients were phoned and then new set of questionnaires with a reminder letter were sent.

#### Reference populations

We used measured with the same instrument for a sample of 851 healthy residents in the Netherlands as a reference population [18].

## Data analysis

### Reference populations

The proportion of peritonitis patients reporting moderate or extreme problems (combined together) on each of the EQ-5D dimensions in the study group was compared to the proportion reported by the general Dutch population using a χ^2 ^tests. Differences in mean EQ-VAS scores were calculated between the study peritonitis patients and the general population stratified by 10-year age groups, and tested for significance using the Student's t-test [26].

Representatively of the sample with HR-QoL measurements for the non-responders (non-respondent analysis) was evaluated using χ^2 ^tests to compare categorical data, and the Student's t-test or the Mann-Whitney U test for continuous data.

### Predictive factors

An initial set of potential factors was based on two previous studies examining factors associated with increased mortality and morbidity in patients with secondary peritonitis [5,13]. These candidate factors were divided into three distinct categories:

1) ***General patient characteristics***: age, gender, and having one or more major comorbidities. Major comorbidities were measured by severity and included cardiovascular disease; chronic obstructive pulmonary disease (COPD); malignancy; renal disease, and diabetes mellitus (DM).

2) ***Peritonitis characteristics***: severity of disease at study entry measured by the APACHE-II score and severity of peritonitis measured by the Mannheim Peritonitis Index (MPI), extent (localized versus diffuse) and type of contamination (clear, turbid, purulent, fecal), etiology of peritonitis (inflammation, perforation, ischemia/necrosis, anastomotic leakage), and community-acquired versus hospital-acquired or nosocomial infection, (these infections include post-operative peritonitis as complication of a previous (elective) surgical intervention or peritonitis that is the result of treatment in a hospital or hospital-like setting)

3) ***Postoperative characteristics***: number of relaparotomies, length of stay in ICU and hospital, duration of mechanical ventilation, complications during ICU stay, i.e., acute respiratory distress syndrome (ARDS). Also factors including having an enterostomy at six months, the number of hospital readmissions (for peritonitis-related morbidity) and experiencing one of the predefined severe morbidities during the six-month follow-up (including incisional hernia, bowel obstruction/herniation, burst abdomen, abdominal compartment syndrome, fistula, intra-abdominal bleeding, perforation, anastomotic leakage, ischemia/necrosis, enterostomy dysfunction, bleeding ulcer, abscess (needing drainage), renal failure, myocardial infarction/embolus/cerebral vascular accident, pneumonia or urosepsis needing readmission (see Appendix 2 for the complete list)).

We used a general linear model to identify factors associated with the EQ-VAS, or with the proportion of patients reporting moderate or severe problems on either of the five dimensions of the EQ-5D. Factors associated with HR-QoL (p <= 0.1) were then entered in a multivariate model, unless predictive factors were strongly correlated with each other, then only one factor with the strongest association was chosen. The functional form of continuous predictors was graphically assessed and, in the case of pertinent non-linearity, a transformation was performed.

Statistical Package for the Social Sciences (SPSS 11.01, SPSS Inc, Chicago, IL) was used for all data analysis.

## Results

A total of 155 surviving patients were eligible for the HR-QoL study and questionnaires were sent to all of them. The overall response rate was 85% (130/155; see Figure 1). The average responses were provided at 6 months and 4 days after index laparotomy.

The mean age of patients at enrollment was 63 years, and 53% of the patients were male (Table 1). Patients at trial entry were generally severely ill, as reflected by a mean APACHE-II score of 15.1 and mean MPI of 19.9 (Table 1).

**Table 1:** General patient, peritonitis and post-operative characteristics (n = 130)

**Patient Characteristics (n= 130)**		**Percentage**
Age; mean (SD)	63 (14)	
Males; n	70	53
≥1 major comorbidity; n	73	56
**Peritonitis Characteristics**		
APACHE – II mean (SD)	15.1 (4.1)	
Mannheim peritonitis index, mean (SD)	19.9 (7.6)	
Extent of contamination:		
1 or 2 quadrants	49	37
Diffuse	82	64
Type of contamination:		
Clear	8	6
Turbid	29	22
Purulent	42	32
Etiology of peritonitis:		
Inflammation	6	5
Perforation	72	55
Ischemia/necrosis	6	5
Anastomotic leakage	41	31
Other	6	5
Hospital-acquired peritonitis patients (peritonitis following earlier elective operation and/or during hospital stay)	69	53
**Postoperative Characteristics**		
Pts with ≥1 relaparotomy n	86	66
Relaparotomies; median (range)	1.0 relaps (1–10)	
Pts admitted to ICU n	115	88
Length of ICU stay; median (P25–P75)	9 days (6–21)	
Patients ventilated n	110	84
Duration of ventilation; median, (P25–P75)	6 days (3–12)	
Length of hospital stay median, (P25–P75)§	34 days (19–60)	
Acute Respiratory Distress Syndrome (ARDS)	7	5.4
Patients readmitted ≥1 at 6 months	74	57
>1 Morbidities during 6-month follow-up*	33	26
Patients with enterostomy at 6 months	73	56

* Information on morbidities missing for one patient (n = 129)

§For two patients the exact hospital stay was unknown, due to transfer to other hospital

There was no significant difference in any patient baseline characteristics; peritonitis characteristics or postoperative characteristics between patients who responded to the HR-QoL questionnaires (n = 130) and patients that did not respond (n= 32) (Figure 1).

### Comparison with other populations

**Figure 2:**
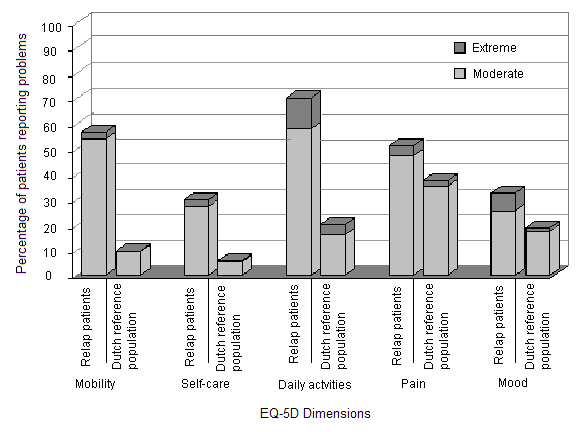
Percentage of HR-QoL problems reported by peritonitis study patients (n = 130) compared to a general reference population from The Netherlands (Dutch reference population) (n = 851) [18] by EQ-5D dimensions.

Compared to a health reference population [18], the peritonitis group reported significantly more problems on all EQ-5D dimensions (p < 0.001 for all dimensions, see Figure 2). Patients with peritonitis showed in all age groups lower EQ-VAS scores than the reference group, indicating worse overall HR-QoL. In the RELAP group, EQ-VAS scores appeared to be low from young till old and did not particularly worsen for those who are older.

### Predictive factors

Results of the univariate analyses evaluating patient, peritonitis and postoperative factors as predictors for HR-QoL at six months are reported in table 2.

**Table 2:** Strength of univariate association between potential predictors and reporting problems on the EQ-VAS and on the EQ-5D on which inclusion in final multivariate model is based.

	**EQ-VAS**	**EQ-5D**					
	
	**VAS**	**Mobility**	**Self-care**	**Daily Activity**	**Pain**	**Mood**	**Included into Multivariate model**
**Patient Characteristics**							
Gender	-	+	-	++	-	-	**Yes**
Age	++	+	-	-	-	++	**Yes**
Major Comorbidity	-	+	-	-	-	+	**Yes**
**Peritonitis characteristics**							
APACHE-II	-	-	-	+	-	-	**No**
Mannheim Peritonitis Index	-	-	-	-	-	-	**No**
Extent of contamination	-	-	-	-	-	-	**No**
Etiology of peritonitis	-	-	-	-	-	-	**No**
Hospital-acquired vs. community-acquired peritonitis	-	-	-	-	-	-	**No**
**Postoperative Characteristics**							
Pts with ≥1 Relaparotomy	-	-	-	-	-	-	**No**
Acute Respiratory Distress Syndrome (ARDS)	-	-	-	+	-	-	**No**
Length of ICU stay	+	++	++	+	-	+	**Yes**
Length of ventilation	+	-	-	+	-	-	**Yes**
Length of hospital stay	++	++	++	++	-	-	**Yes**
Readmissions during follow-up	-	-	-	-	-	-	**No**
Severe morbidity during 6 month follow-up (Appendix 2)	+	-	-	+	-	+	**Yes**
Enterostomy at 6 months	+	++	+	+	-	-	**Yes**

+ Univariate significance (p < 0.10), ++ univariate significance < 0.05

- No univariate significance found

#### General patient characteristics

In a univariate analysis men reported significantly fewer problems with mobility and daily activities. Increasing age decreased overall well-being and increased problems in mobility, but was protective for mood problems with younger patients scoring more mood problems. Major comorbidities at baseline were predictive for more problems related to mobility and mood at six months (Table 2).

#### Peritonitis characteristics

Peritonitis characteristics were not associated with scores on EQ-VAS or EQ-5D when looking at severity of disease or peritonitis severity, etiology or type and extent of the contamination (Table 2). There were no HR-QoL differences between patients with community-developed peritonitis and patients with hospital-acquired peritonitis.

#### Postoperative characteristics

Patients who stayed longer in ICU and/or surgical hospital-ward reported more problems on all functional impairment dimensions mobility, self-care and daily activities and overall well-being, but not on the pain and mood dimensions (Table 2). Although ICU stay and hospital stay are clearly associated with HR-QoL, whilst in a univariate analysis mechanical ventilation was not.

Readmissions during the six-month follow-up were also associated with lower HR-QoL scores. Patients who still had an enterostomy six-months following surgery reported more problems in the functional impairment dimensions: mobility, self-care and daily activities (the combination these dimensions is often referred to as a specific discipline within HR-QoL called activities in daily life or ADL). Overall those patients reported more well-being problems than patients without an enterostomy (Table 2).

#### Multivariate analysis

The following factors were entered in the multivariate analysis based on the results of the univariate association (p ≤ 0.10) with HR-QoL with at least two of the five EQ-5D dimensions or an effect on the EQ-VAS (Table 2): gender, major comorbidity, enterostomy at six months, length of ICU stay and length of hospital stay (a log2 transformation was done to create linearity) and severe morbidity during follow-up. From the literature, it was decided that age should always be added to the models, irrespective of the univariate analyses [22,28-30]. ICU stay and hospital stay were highly correlated (Spearman's R = 0.681) and therefore not both of the factors could be added to the multivariate model. Length of hospital stay was selected to best represent the accumulation of what a patient underwent following secondary peritonitis, used as an adequate proxy for poor patients recovery and potential complications. The same set of factors were included in all six models (the EQ-VAS: Table 3 and the five EQ-5D dimensions: Table 4).

**Table 3:** Impact of potential predictors on EQ-VAS scores. Results expressed as absolute changes in mean scores derived from multivariate model.

	**Euroqol Visual Analogue Scale (n = 127‡)**	
	
	**Mean difference in EQ-VAS score†**	**P-value**
Gender (Male vs. females)	4.0	0.193
Age (per 10 years increase)	-2.9	0.348
Patients without major comorbidity at study entry	3.9	0.192
**Every doubling of the length of hospital stay**	**-3.8**	0.015*
Patients without severe morbidity during six month follow-up	6.4	0.077
Patients with no enterostomy at six months	4.9	0.125

* Significant p < 0.05, **bold**

‡ Three patients were dropped due to missing VAS scores

† Lower EQ-VAS scores indicate poorer health status

**Table 4:** Odds ratios for reporting moderate/severe problems on each of the dimensions of the EQ-5D. Results from multivariate model including all listed factors

**Predictive Factors:**	**Mobility (n = 128)**		**Self-care (n = 129)**		**Daily Activities (n = 129)**		**Pain/Discomfort (n = 129)**		**Mood (n = 130)**	
	OR	P-value	OR	P-value	OR	P-value	OR	P-value	OR	P-value

**Gender (Female)**	**2.9**	0.013*	1.5	0.296	**3.7**	0.006*	**2.3**	0.030*	1.7	0.176
**Age (per 10 years increase)**	1.0	0.246	0.99	0.534	0.98	0.148	0.74	0.078	**0.54**	<0.001*
**Patients with major comorbidity at study entry**	2.0	0.120	0.92	0.848	1.1	0.782	1.8	0.151	**3.6**	0.007*
**Every doubling of the length of hospital stay**	**1.6**	0.020*	**2.5**	<0.001*	**1.9**	0.010*	1.1	0.537	0.91	0.649
**Patients with severe morbidity during six month follow-up**	0.71	0.484	0.58	0.294	0.83	0.719	2.4	0.065	2.0	0.130
**Patients with an enterostomy at six months**	**2.8**	0.016*	1.7	0.240	**2.8**	0.027*	1.2	0.613	1.5	0.320

Significant p < 0.05, **bold**

Patients report moderate and/or severe problems

In the multivariate analysis the only independent factor that was predictive for poorer worse overall patient well-being, as measured by the EQ-VAS, was length of hospital stay (log2 transformed); every doubling of the length of hospital stay decreased the EQ-VAS (0–100) score by 3.8 points (p = 0.015, Table 3).

In the logistic models for each dimension of the EQ-5D the following factors were predictive of HR-QoL (Table 4). Females reported more mobility problems (OR = 2.9, p = 0.013), more problems in daily activities (OR = 3.7, p = 0.006) and more pain and discomfort (OR = 2.3, p = 0.037). Increasing age was associated with fewer problems with mood (OR = 0.54 per 10 years, p < 0.001); whilst patients with a major comorbidity were more likely to report problems on the mood dimension (OR = 3.6, p = 0.007).

Length of hospital stay was associated with more problems in all ADL dimensions; a doubling of the length of hospital stay increased problems in mobility (OR = 1.6, p = 0.02), self-care (OR = 2.5, p < 0.001) and daily activities (OR = 1.9, p = 0.01). Whilst severe morbidity (as experienced) during the six months follow-up was no longer independently associated with lower HR-QoL in the multivariate model. However, longer hospital stay is in part due to severe morbidity; so clinically it may not be possible to consider them apart.

Patients with an enterostomy at six-month follow-up reported more problems for mobility (OR = 2.8, p = 0.016) and daily activities (OR = 2.8, p = 0.027), but not for self-care or mood.

## Discussion

This study shows that patients treated for secondary peritonitis report considerably more complaints on all EQ-5D dimensions six months after initial surgery than a general reference population. Furthermore, HR-QoL at six months was found to be associated with several patient characteristics and particularly postoperative characteristics, whereas factors directly related initial severity of peritonitis did not affect HR-QoL. [11].

### Comparisons with other populations

The comparison with a general reference population of healthy individuals allows for a better understanding of the extent of reduction in HR-QoL in this patient group. To give an even better perspective of the extent of the HR-QoL presented here we can compare our peritonitis patient group to a group of general sepsis patients, who were also measured at 6 months following ICU discharge using the Euroqol questionnaire [11]. Comparing these groups shows our peritonitis patients reported more problems with ADL, e.g. more problems with mobility and daily activities, despite having comparable APACHE-II scores, hospital stay and length of ICU stay with the general sepsis patients. This difference in ADL dimensions could, at least in part, be explained by some extent of disfiguration and protracted wound healing following major surgery for patients with peritonitis in contrast to patients with sepsis (resulting from other causes). As well, the peritonitis patients often have an enterostomy for a lengthy period of time, which in this study has also been shown to reduce patients' mobility and daily activities. In contrast, secondary peritonitis patients reported fewer mood problems than patients with sepsis from other causes. One possible factor that could account for this difference is the higher mean age of our peritonitis population. In this study and an earlier retrospective study we have shown that older secondary peritonitis patients report fewer mood problems [11,31].

### Factors associated with lower HR-QoL

In our study, general patient characteristics played an important role in the HR-QoL at 6 months follow-up. Female patients were more likely to report problems with overall HR-QoL, mobility, daily activities, pain and discomfort and mood. Of the nine studies, involving survivors of critical illness and intensive care patients as reviewed by Dowdy et al. [20], associations between HR-QOL and gender were found in only two studies [32,33].

Peritonitis patients showed a clear association between increased age and improved emotional health, possibly related to an adjusting process. Similar findings were reported in other studies, showing that elderly patients demonstrated more positive health attitudes than younger survivors [11,22,30,31]. However, in a recent review no significant associations were found between age and mental health (SF-36), anxiety/depression (EQ-5D) and/or psychosocial QoL [20].

Comorbidity, often an important determinant of health outcomes, was frequently present in this patient group. Patients with 1 or more major comorbidity reported significantly more moods problems. In these analyses we only considered major comorbidities, indicating a pre-existent more severely compromised clinical condition. Although, most patients also suffered from an underlying disease (i.e., primary condition) or underwent a primary procedure prior to their secondary peritonitis, these factors were not considered in major comorbidities. Primary conditions are more likely to be the actual cause or part of the etiology of the peritonitis than is an actual comorbidity; these included malignancy, diverticulitis, Crohn's disease, ulceritis and colitis ulcerosa. Disease severity measure by the APACHE-II has been shown to be an adequate predictor for survival in abdominal sepsis patients [2,5,33-35]. Studies relating disease severity with HR-QOL studies have found mixed results; in some papers preoperative severity of disease was a predictor of HR-QoL [30,33,36-40], whilst others observed no correlation [28,29,41-43]. In our study higher APACHE scores were not associated with poorer HR-QoL. This absent relation could be explained by the homogeneity of the sample with respect to disease severity; only APACHE-II scores higher than 10 were included in the study, reflecting severe illness with an expected mortality around 30% [35]. In this spectrum of severe illness the variability in APACHE-II might be insufficient to predict future HR-QoL.

We found no relation between initial peritonitis severity (MPI), extent or type of contamination and the etiology of the peritonitis and HR-QoL at six months. This indicates that the HR-QoL outcome of the most severe peritonitis patients may in some cases be far better than anticipated during the initial phase. For example, if a peritonitis patient is admitted to the ICU with a high MPI score and has a diffuse fecal peritonitis then, conditional on survival, their HR-QoL at 6 months follow-up may be similar to those patients that were admitted with less severe peritonitis. This indicates that although these factors are indicators of mortality and morbidity, by themselves they are not associated with poorer HR-QoL at 6 months. As well, HR-QoL differences were not found between patients with community-developed peritonitis and patients with nosocomial peritonitis.

In this study the strongest factor associated with lower HR-QoL was length of hospitalization. This suggests that an extended and more complicated course of disease with longer ICU stay combined with severe morbidity accumulates into worse quality of life, most notably in problems with mobility, self-care, and daily activities. ADL problems were primarily related to an extended course of disease encountered during the hospital stay with longer ICU stay – likely related to an accumulation of factors, for example more severe organ dysfunction, such as ARDS, MOF, septic shock and critical illness neuropathy and depended on the patients' response to peritonitis – rather than the underlying etiology and extent of the peritonitis at presentation.

Contrary to expectations, experiencing disease-related morbidity during the six-month follow-up on its own was not an independent predictor for EQ-5D or EQ-VAS outcomes. This is partially due to the multivariate nature of our analysis, where length of hospital probably includes ICU stay in what it measures. Findings in the literature on the relation between length of hospital or length of ICU stay and HR-QoL vary: some studies also found that length of stay was strongly related to HR-QoL [22,29,30,33,40], while other studies found no relation [20,44].

Another particular sequel of the disease is having an enterostomy constructed at surgery for peritonitis, which in these patients is usually still present at six months follow-up. As expected, patients with an enterostomy reported more problems with mobility and daily activities. Reduction of the length of time until restoring continuity in those with a temporary enterostomy, as well as being more liberal with primary anastomosis in some situations (i.e., diverticulitis) may improve long-term HR-QoL.

We assessed HR-QoL using a generic questionnaire, which enabled us to make comparisons to the general population and other diseases populations [22]. The EQ-5D and EQ-VAS have also been recommended as the choice of generic HR-QoL patient groups, and well validated. Nonetheless, applying a disease-specific questionnaire, including peritonitis specific symptoms and complaints, may allow for more insight into possible factors that may not be detected by a generic HR-QoL instrument [24,45].

It may be a viable option that hospitals consider investing into a tailored support network for patients with more lengthy hospitalization stays, to better prepare both the patient and the home caregivers for the period following discharge characterized by diminished HR-QoL. Younger patients of working age and patients with existing major comorbidity seem to warrant more psychosocial support when discharged from the hospital, which could in turn enable them to return to the workforce more quickly and reduce costs due to loss of productivity. Once the acute life-threatening situation has dissipated and patients are in the surgical ward or have been discharged there may be ample opportunities to consider the patients' psychosocial network. The results also suggest that this support should be aimed at all peritonitis patients, irrespective of their severity of illness at presentation, since their at six months HR-QoL is not different from those with a seemly more favorable presentation, as severity of peritonitis is not an important indicator of later HR-QoL.

## Authors' contributions

MB, KB, CdB andOvR conceived the study. KB and OvRcoordinated the study. JB and MS participated in its design and aided in the statistical analysis. BO, MS and EAR participated in the coordination and data analysis. MB, OvR, CM, HG, PdG, EH, MG and EPS were responsible for patient inclusion. All authors read and approved the final manuscript. The Dutch Peritonitis Study Group (Appendix 1) participated in the design and coordination of the study and was responsible for patient inclusion.

## Appendix 1

RELAP trial clinical centers and investigators of the Dutch Peritonitis Study Group (from the Department of Surgery, Academic Medical Center Amsterdam, The Netherlands).

All investigators are from Departments of Surgery unless specified (E) Clinical Epidemiology and Biostatistics (E) or (I) Intensive Care or Medical Psychology (MP).

O van Ruler, KR Boer (E), JB Reitsma (E), CW Mahler, EA Reuland, JWO van Till, BC Opmeer (E), PMM Bossuyt (E), MJ Schultz (I), MA Sprangers (MP), H Obertop, DJ Gouma, CAJM de Borgie (E), MA Boermeester, Academic Medical Center, Amsterdam; EPh Steller, P. Tanis, H Hart (I), St Lucas Andreas Hospital, Amsterdam; MF Gerhards, M Guijt, HM Oudemans (I), Onze Lieve Vrouwe Gasthuis, Amsterdam; K. Bosscha, E Ritchie, M Vermeer, Bosch Medical Centre, Den Bosch, The Netherlands. PW de Graaf, B van Etten, C Haazer, E Salm (I), Reinier de Graaf Hospital, Delft; B Lamme, EJ Hesselink, H Rommes (I), Gelre Hospital, Apeldoorn; RJ Oostenbroek, L te Velde, G Govaert, HH Ponssen (I), Albert Schweitzer Hospital, Dordrecht; HG Gooszen, MK Dinkelman, LPH Leenen (I), University Medical Centre Utrecht; EGJM Pierik, KWW Lansink, J Bakker (I), Isala Clinics, Zwolle;

Key staff and steering committee at coordinating center (AMC Amsterdam) RELAP trial:

O van Ruler (study coordinator and investigator), EA Reuland (data management), CW Mahler (investigator), JB Reitsma (epidemiologist), CAJM de Borgie (epidemiologist), KR Boer (quality of life investigator), BC Opmeer (economist), MA Boermeester (surgeon, supervisor, project leader) from the Department of Surgery, Academic Medical Center Amsterdam, The Netherlands.

## Appendix 2: Disease-related morbidity

Non-surgical or conservative treatment of:

• **Fistula **(non-anatomical connection between hollow organ and cutis or between two hollow organs)

• **Wound dehiscence/incisional hernia **(full thickness discontinuity in abdominal wall with bulging of abdominal content) **with obstruction**

• **Abscess needing percutanous drainage **(pus-containing non-pre-existing cavity confirmed by positive Gram-stain or culture)

• **Renal failure **(urine production < 500 cc/24 h with rising level of blood urea and creatinin) **combined with dehydration **(decreased circulating volume with raised hematocrit needing intravenous rehydration) based on inadequate oral intake and/or nausea/vomiting. Only when needing readmission.

• **Myocardial infarction **(ECG and enzyme changes being suggestive of MI, needing admission to CCU) or **pulmonary embolus**(ventilation perfusion mismatch on lung scintigraphy) or **cerebrovascular accident **(ischaemic or non ischaemic with persistent paresis or paralysis without previous history

• **Gastric or duodenal bleeding **(needing endoscopic treatment or embolisation therapy)

• **Respiratory failure **(due to pneumonia, pleural effusion or pulmonary edema needing oxygen therapy or mechanical ventilation)

• **Urosepsis **(urinary tract infection with positive urine and blood cultures and circulatory shock)

**Surgical **intervention for disease-related morbidity

• **Incisional hernia **(full thickness discontinuity in abdominal wall with bulging of abdominal contents with or without obstruction with disabling complaints interfering with daily activities, needing surgery)

• **Bowel obstruction or herniation due to intra-abdominal adhesions **(diagnosis must be confirmed during surgery)

• **Burst abdomen **(complete midline or transverse discontinuity in abdominal wall)

• **Abdominal compartment syndrome **(intra-abdominal hypertension >25 mmHg with tense abdomen and with increasing respiratory and/or renal failure; measured by the urinary bladder pressure method (modified Burch criteria)

• **Fistula **(non-anatomical connection between intestine and cutis or between two hollow organs needing surgery)

• **Intra-abdominal bleeding **(Only when septic bleeding after index laparotomy or relaparotomy or when surgical bleeding after relaparotomy but not after index laparotomy)

• **Intraabdominal haematoma **(needing surgical evacuation)

• **Perforation **(of visceral organ; confirmed at surgery)

• **Anastomotic leakage **(anastomotic leak on contrast imaging needing surgery or contrast enhanced CT confirmed at relaparotomy)

• **Ischemia or necrosis of a visceral organ **(critically reduced blood flow to an intra-abdominal organ causing tissue loss; confirmed at pathological examination)

• **Enterostomy dysfunction **(due to prolaps, stenosis or retraction)

• **Gastric or duodenal ulcer bleeding **(needing intervention of any type)
